# Brusatol inhibits metastasis of triple-negative breast cancer through metabolic reprogramming

**DOI:** 10.3389/fonc.2026.1708878

**Published:** 2026-02-27

**Authors:** Xuan Yu, Xueling Diao, Xiaokai Fan, Chuantao Wu, Siyuan Wang, Maoxuan Liu, Edwin Cheung, Liang Chen

**Affiliations:** 1 Shenzhen Laboratory of Tumor Cell Biology, Institutes of Biomedicine and Biotechnology, Shenzhen Institutes of Advanced Technology, Chinese Academy of Sciences, Shenzhen, China; 2 University of Chinese Academy of Sciences, Beijing, China; 3 Greater Bay Area Institute of Precision Medicine (Guangzhou), School of Life Sciences, Fudan University, Shanghai, China; 4 Molecular Cancer Research Center, School of Medicine, Shenzhen Campus of Sun Yat-Sen University, Sun Yat-Sen University, Shenzhen, China; 5 College of Pharmacy, Shenzhen Technology University, Shenzhen, China; 6 Cancer Centre, Faculty of Health Sciences, University of Macau, Taipa, Macau, China

**Keywords:** breast cancer, Brusatol, metabolic reprogramming, natural compound, pentose phosphate pathway

## Abstract

**Background:**

Triple-negative breast cancer (TNBC) is an aggressive subtype of breast cancer (BC) characterized by a high risk of metastasis and poor prognosis. Current chemotherapy-based treatments are often limited by systemic toxicity and drug resistance. Brusatol (BRU), a natural compound with reported anti-tumor activity and low toxicity, has not been explored in the context of cancer metastasis or metabolic reprogramming. This study aimed to uncover the anti-metastatic mechanism of BRU by targeting the metabolic adaptation of detached TNBC cells.

**Methods:**

The suppressive effect of BRU was assessed via LDH release assays, trypan blue staining, tumor spheroid culture and spontaneous metastasis assays. To elucidate the underlying mechanisms, global metabolomics analysis, NADPH/NADP^+^ measurements, intracellular ROS detection by flow cytometry, and quantitative PCR for metabolic gene expression were applied to evaluate metabolic alterations and redox imbalance.

**Results:**

BRU promoted membrane damage and cell death in extracellular matrix (ECM)-detached TNBC cells and suppressed metastasis *in vivo*. Metabolomics analysis showed that BRU inhibited metabolic pathways, including the pentose phosphate pathway (PPP), glycolysis, and the tricarboxylic acid (TCA) cycle, while significantly reducing NADPH levels and exacerbating redox stress.

**Conclusions:**

These findings suggest that BRU targets metabolic plasticity in TNBC cells, highlighting its potential as an adjuvant therapy to enhance anti-tumor efficacy while reducing chemotherapy-associated toxicity.

## 1 Introduction

Breast cancer is the most prevalent cancer and the leading cause of cancer-related mortality among women worldwide (1). Among its subtypes, triple-negative breast cancer (TNBC) is a highly aggressive form, accounting for 15%–20% of all breast cancers, and is associated with a high risk of distant metastasis. The metastatic progression of TNBC involves a series of steps, including the detachment of tumor cells from the extracellular matrix (ECM), local invasion and migration, intravasation into blood or lymphatic vessel, survival in circulation and eventual colonization of secondary tumors at distant sites (2, 3). Loss of attachment to the ECM and neighboring cells triggers a form of programmed cell death known as anoikis (4). To survive in the bloodstream and establish metastases in distant organs, tumor cells must develop resistance to anoikis, enabling anchorage-independent growth (5). In addition to anoikis resistance, ECM detachment induces a range of anoikis-independent changes, such as altered nutrient uptake (6–8), metabolic reprogramming (9–11), mitophagy induction (12), and rearrangement of signal transduction pathways (13–15). These changes can compromise cell viability and even lead to cell death (16, 17). Since TNBC cells frequently encounter ECM detachment during tumor progression and metastasis, eliminating ECM-detached cells is crucial for improving the survival rate of patients with advanced TNBC (18).

Tumor metabolic reprogramming refers to adaptive alterations in metabolic pathways that cancer cells undergo in response to environmental or pathological conditions, which helps to meet the demands of anabolic growth and fuel tumor development (19). For instance, ECM-attached tumor cells preferentially utilize glycolysis as their primary glucose metabolic pathway, even under normoxic conditions, to rapidly generate energy for tumor growth (20). In contrast, ECM-detached TNBC cells exhibit decreased glucose uptake (6) and elevated levels of reactive oxygen species (ROS) (7, 21). Under these conditions, the pentose phosphate pathway (PPP) may replace glycolysis as the dominant pathway (22), generating NADPH to maintain redox homeostasis (16), which enables ECM-detached TNBC cells to evade death. Consequently, targeting metabolic reprogramming represents a promising therapeutic avenue for cancer treatment (23).

In recent years, natural products have gained attention as promising agents for cancer therapy due to their accessibility, low cost, minimal toxicity, limited side effects, reduced likelihood of drug resistance, and high efficacy (24). Brucea javanica oil, extracted from the seeds of *Brucea javanica*, has been used to treat various diseases, including cancer, amoebic dysentery, and malaria. Brusatol (BRU), a homolactone compound purified from Brucea javanica oil, is the primary active component of the oil and has demonstrated significant anticancer activity (25). Studies have shown that the anticancer effects of BRU include promoting the degradation of the transcription factor Nrf2, suppressing downstream antioxidant gene expression, inducing apoptosis, and inhibiting tumor cell proliferation, epithelia-mesenchymal transition (EMT), and invasion (26–29). Previous research (30) reported that low-dose combination therapy with BRU and polydatin significantly inhibited the growth of TNBC cells while minimizing toxic side effects. Furthermore, BRU has been shown to reverse chemoresistance in various tumor cells, sensitizing them to chemotherapeutic agents and enhancing the efficacy of chemotherapy, including combination with paclitaxel in TNBC treatment (31). However, most studies on BRU have been conducted on ECM-attached cells, with limited research exploring its effects on tumor metabolism and distant metastasis.

In this study, we established that BRU suppresses TNBC metastasis by inducing profound metabolic reprogramming. Mechanistically, BRU downregulated PPP flux, reduced NADPH production, and promoted cell death in ECM-detached TNBC cells *in vitro*. *In vivo*, BRU significantly suppressed the colonization of ECM-detached tumor cells and inhibited their growth in mouse models. These findings provide new insights into the therapeutic potential of BRU and suggest a novel direction for the treatment of TNBC.

## 2 Materials and methods

### 2.1 Reagents

Brusatol (HY-19543, purity 99.89%) was purchased from MedChemExpress (Monmouth Junction, NJ, USA). DMSO (ST038), Trypan Blue, DCFH-DA (2’, 7’-dichlorodihydrofluorescein diacetate), Cell Counting Kit-3D and the NADP^+^/NADPH Assay Kit with WST-8 were obtained from Beyotime Biotechnology (Shanghai, China). DMEM was sourced from VivaCell (Shanghai, China), and fetal bovine serum (FBS) was obtained from TransGen Biotech (Beijing, China). Poly (2-hydroxyethyl methacrylate) (poly-HEMA) was purchased from Sigma-Aldrich (St. Louis, MO, USA). The CyQUANT™ LDH Cytotoxicity Assay Kit was purchased from Thermo Fisher Scientific (Rockford, IL, USA). The DCFH-DA and D-Luciferin sodium salt was obtained from Yeasen Biotechnology (Shanghai, China). The SYBR Green PCR Master Mix (Q712-03) was purchased from Vazyme (Nanjing, China).

Commercial antibodies used in this study included G6PD, β-actin, and HRP-conjugated Affinipure goat anti-rabbit/mouse IgG (H+L) secondary antibodies, all obtained from Proteintech (Wuhan, China).

### 2.2 Cell culture

Human breast cancer MDA-MB-231 cells (CL-0150) were purchased from Procell Life Science & Technology Co., Ltd. (Wuhan, China). Human breast cancer BT-549 cells (STCC10511P-1) were purchased from Servicebio (Wuhan, China). Cells were cultured in DMEM supplemented with 10% FBS and 1% penicillin-streptomycin (PS) (VivaCell, Shanghai, China) at 37°C in a humidified atmosphere of 5% CO_2_.

For matrix-detached culture, poly-HEMA (1.2% w/v) was dissolved in 95% ethanol and incubated overnight at 37°C on a shaking platform. Cell culture plates were coated with the poly-HEMA solution, dried under sterile conditions, and seeded with cells at equivalent densities for matrix-attached and matrix-detached experiments.

### 2.3 Western blot

MDA-MB-231 cells were seeded in 12-well plates or poly-HEMA-precoated plates and treated with DMSO or BRU for 12 hours. Cells were washed twice with precooled PBS, lysed with SDS lysis buffer (containing a protease inhibitor cocktail), and collected into EP tubes. Protein samples were mixed with 5× loading buffer and heated at 95 °C for 10 minutes.

Proteins were separated by 10% sodium dodecyl sulfate-polyacrylamide gel electrophoresis (SDS-PAGE) and transferred onto 0.45 μm polyvinylidene fluoride (PVDF) membranes (Millipore, Bedford, MA, USA). Membranes were blocked in 5% non-fat milk for 2 hours at room temperature, washed with PBST (PBS containing 0.1% Tween-20), and incubated with primary antibodies overnight at 4 °C. After washing three times with PBST (5 minutes each), membranes were incubated with secondary antibodies for 1 hour at room temperature and washed again three times with PBST.

Protein signals were detected using enhanced chemiluminescence (ECL, Yeasen, Shanghai, China) and visualized with an Amersham Imager AI600 (Cytiva, Washington, DC, USA). Gray values of protein bands were quantified using ImageJ software.

### 2.4 Global metabolomic analysis

The extraction, detection, and quantitative analysis ofmetabolites in the samples were performed by Metware Biotechnology (Wuhan, China). Briefly, cells from each group were resuspended in 100 μL of purified water. A 50 μL aliquot of the cell suspension was mixed with 200 μL of precooled methanol and vortexed for 2 minutes. The mixture was subjected to three freeze–thaw cycles (frozen in liquid nitrogen for 5 minutes, thawed on ice for 3 minutes, and vortexed for 2 minutes). After the final cycle, the supernatants were collected by centrifugation at 12, 000 × g for 10 minutes at 4 °C. The supernatants were further incubated at -20 °C for 30 minutes and centrifuged again at 12, 000 × g for 10 minutes. A 180 μL aliquot of the supernatant was deproteinized using a protein precipitation plate and analyzed by high-performance liquid chromatography–mass spectrometry (LC–MS). The solvent system consisted of ultrapure water with 10 mM ammonium acetate and 0.3% ammonium hydroxide (A) and 90% acetonitrile/water (B). The gradient elution started at 95% B (0–1.2 min), decreased to 70% B (8 min), then to 50% B (9–11 min), and returned to 95% B (11.1–15 min).

Mass spectrometry data were initially processed using Analyst 1.6.3 software and subsequently refined with MultiQuant 3.0.3. Chromatographic peaks corresponding to the target analytes across samples were integrated and corrected against the retention time and peak profiles of reference standards to ensure accurate qualification and quantification. A series of standard solutions was prepared at the following concentrations: 0.01, 0.02, 0.05, 0.1, 0.2, 0.5, 1, 2, 5, 10, 20, 50, 100, 200, 500, 1000, 2000, 5000, 10000, and 15000 ng/mL. For NADPH and D(+)-Glucose, the concentration points were 0.1, 0.2, 0.5, 1, 2, 5, 10, 20, 50, 100, 200, 500, 1000, 2000, 5000, 10000, 20000, 50000, 100000, and 150000 ng/mL. The chromatographic peak intensity corresponding to the quantitative signal was recorded for each concentration. Standard curves were constructed by plotting the external standard concentration on the x-axis against the ratio of the external standard peak area to the internal standard peak area on the y-axis. The peak areas from experimental samples were then applied to the linear regression equation of the corresponding standard curve to determine their concentrations. To express the metabolite content normalized to cell number, the following formula was applied:


m=(c×v)/(n×1000)

where: m is the metabolite concentration (ng per million cells), c is the concentration (ng/mL) derived from the standard curve based on the sample peak area; v is the volume of extraction solution (µL); n is the amount of sample transferred (million cells). To further compare metabolite abundance in DMSO and BRU groups, we normalized metabolite abundance by using Z-score transformation to remove scale differences between variables. The metabolite abundance were visualized as a heatmap using the ComplexHeatmap (version 2.7.1.1009) package in R (version 4.4.3).

### 2.5 NADP^+^ and NADPH levels and ratio

NADP^+^ and NADPH levels were measured using the NADP^+^/NADPH Assay Kit with WST-8 (S0179, Beyotime, China) according to the manufacturer’s protocol. Briefly, 1 × 10^6^ cells were lysed and centrifuged at 12, 000 × g for 10 minutes at 4 °C. The supernatant was divided into two parts: one portion was heated at 60°C for 30 minutes to deplete NADP^+^ (leaving only NADPH), while the other portion was kept on ice as the unheated sample (containing both NADPH and NADP^+^). After cooling, both samples were incubated with the working solution at 37 °C for 10 minutes to allow NADP^+^ to convert into NADPH. The developer was added, and the reaction was incubated for 40 minutes to form formazan, which was then measured at 450 nm. NADP^+^ and NADPH levels were calculated using an NADPH standard curve and normalized to the total protein content of each sample.

### 2.6 ROS measurements

MDA-MB-231 or BT-549 cells were treated with 20 nM BRU or DMSO under ECM-attached or ECM-detached conditions for 12 hours. Cells were collected, incubated with 10 μM DCFH-DA for 30 min, washed with PBS, and re-harvested. DCF fluorescence was immediately measured using the Cyto FLEX flow cytometer (Beckman, Calif., USA) and analyzed by FlowJo software.

### 2.7 Quantitative real-time PCR

After treatment with 20 nM BRU or DMSO under ECM−attached or ECM−detached conditions for 12 h, cells were collected for RNA extraction. Total RNA was extracted with RNAex Pro RNA Reagent (Accurate Biology, Guangzhou, China) following the manufacturer’s protocol. RNA (2 μg) was reverse-transcribed with the PrimeScript RT Reagent Kit with gDNA Eraser (TaKaRa, Tokyo, Japan). Quantitative real-time PCR was performed on the CFX96 Real-Time System (Bio-Rad, Calif., USA) using SYBR Green PCR Master Mix (Vazyme, Nanjing, China). β−Actin served as the endogenous reference gene, and all reactions were run in triplicate. The threshold cycle (Ct) values were determined, and relative mRNA expression was calculated using the 2−ΔΔCt method. Statistical analysis and graphing were performed with GraphPad Prism 9 software (GraphPad Software Inc., San Diego, CA, USA).The primer sequences are as follows:

β-Actin(forward:ATTGGCAATGAGCGGTTCCG; reverse:AGGGCAGTGATCTCCTTCTG),

HK2 (forward:GATTGCCTCGCATCTGCTTG; reverse:GCTCCAAGCCCTTTCTCCAT),

G6PD (forward:CGAGGCCGTCACCAAGAAC; reverse:GTAGTGGTCGATGCGGTAGA),

TKT (forward:TCCACACCATGCGCTACAAG; reverse:CAAGTCGGAGCTGATCTTCCT),

6PGD (forward:GACATCATCATTGACGGAGGAAA; reverse:GGGCCACGCTTCTTTGTTC),

PFKFB (forward:CAGTTGTGGCCTCCAATATC; reverse:GGCTTCATAGCAACTGATCC),

GAPDH (forward:ACAACTTTGGTATCGTGGAAGG; reverse:GCCATCACGCCACAGTTTC),

PGK1 (forward:TTAAAGGGAAGCGGGTCGTTA; reverse:TCCATTGTCCAAGCAGAATTTGA),

PGAM1 (forward:GTGCAGAAGAGAGCGATCCG; reverse:CGGTTAGACCCCCATAGTGC),

ENO1 (forward:TGGTGTCTATCGAAGATCCCTT; reverse:CCTTGGCGATCCTCTTTGG),

PKM2 (forward:ATGTCGAAGCCCCATAGTGAA; reverse:TGGGTGGTGAATCAATGTCCA),

LDHA (forward:ATGGCAACTCTAAAGGATCAGC; reverse:CCAACCCCAACAACTGTAATCT),

### 2.8 Cell death assay

Cell viability was assessed using the Trypan Blue Staining Kit (C0011, Beyotime, China) according to the manufacturer’s instructions. After treatment, cells were collected and washed with PBS. The cell suspension was mixed with an equal volume of trypan blue and incubated for 5 minutes. Stained cells were placed on a hemocytometer, and multiple fields were imaged under a bright-field microscope. The number of live and dead cells was calculated, and cell mortality was determined using the formula: Cell death (%) = dead cells/(dead cells + live cells). Each experiment was performed in triplicate.

### 2.9 LDH assay (lactate dehydrogenase)

Cytotoxicity was evaluated using the LDH Cytotoxicity Assay Kit (Thermo Fisher Scientific) according to the manufacturer’s protocol. Briefly, 5, 000 cells were cultured in 96-well plates (adherent or poly-HEMA-coated) for 48 hours in the presence of DMSO or BRU (20 or 100 nM). Forty-five minutes before the end of treatment, 10× lysate was added to the maximum LDH group, while sterile ultrapure water was added in other groups to maintain equal volumes. Following centrifugation at 400 × g for 5 minutes, 50 μL of cell culture supernatant was transferred to a new 96-well plate. Subsequently, 50 μL of reaction mixture was added to each well and incubated for 30 minutes at room temperature. Absorbance was measured at 490 nm and 680 nm using a microplate reader, and LDH activity was calculated accordingly.

### 2.10 Tumor spheroid culture

Approximately, 4×10^3^ MDA-MB-231 or BT549 cells were then seeded in PrimeSurface 96V plates (MS-9096VZ, S-Bio, Japan) and cultured for 6 days at 37 °C with 5% CO2. The growth of the spheroids was observed under a bright-field microscope every day. The cell viability was determined by the Cell Counting Kit-3D reagent.

### 2.11 Mouse xenograft models

BALB/c nude mice (female, 5 weeks old) were purchased from Beijing Vital River Laboratory Animal Technology Co., Ltd. MDA-MB-231-Luc cells (1 × 10^6^ cells in 100 μL PBS) were injected into the tail vein of mice. Mice were randomly divided into two groups (n = 3 per group, No animals were excluded) and treated intraperitoneally with PBS (containing 1% DMSO) or Brusatol (2 mg/kg). Treatments were administered on days 0, 5, 10, 15, 20, and 25, in a 5-day repeating cycle for six cycles. Bioluminescence imaging was performed on days 1, 10, 20, and 30. Briefly, mice were intraperitoneally administered D-luciferin, anesthetized with isoflurane 10 minutes later, and then imaged using an IVIS Lumina III Imaging System (PerkinElmer, USA), three pictures were taken and analyzed per mouse. At the experimental endpoint, mice were euthanized by cervical dislocation, and lungs were harvested for *ex vivo* imaging with the same system. All animal experiments were approved by the Institutional Animal Care and Use Committee (IACUC) at SIAT.

Excised lungs were fixed in 4% paraformaldehyde (PFA) and embedded in paraffin. Tissue sections were prepared and stained with hematoxylin and eosin (H&E) for histological analysis. Metastatic lung nodules in H&E-stained sections were identified by their characteristic morphology. Tumor nodules and tumor areas in each lung section were quantified using CaseViewer software (3DHISTECH). The average number of tumor nodules and total tumor area per lung section were calculated to assess tumor burden.

### 2.12 Statistical analysis

All data are presented as the mean ± SD values from at least three independent experiments (n ≥ 3). Statistical comparisons were performed using an unpaired two-tailed Student’s t-test or two-way ANOVA, as specified in the figure legends. All statistical analyses were conducted using GraphPad Prism software. The normality of data distribution was assessed using the Shapiro-Wilk test. Significance levels are denoted as *p< 0.05, **p< 0.01, and ***p< 0.001, ns, no significance.

## 3 Result

### 3.1 Brusatol promotes ECM-detached cell death in TNBC cells

**Figure 1:**
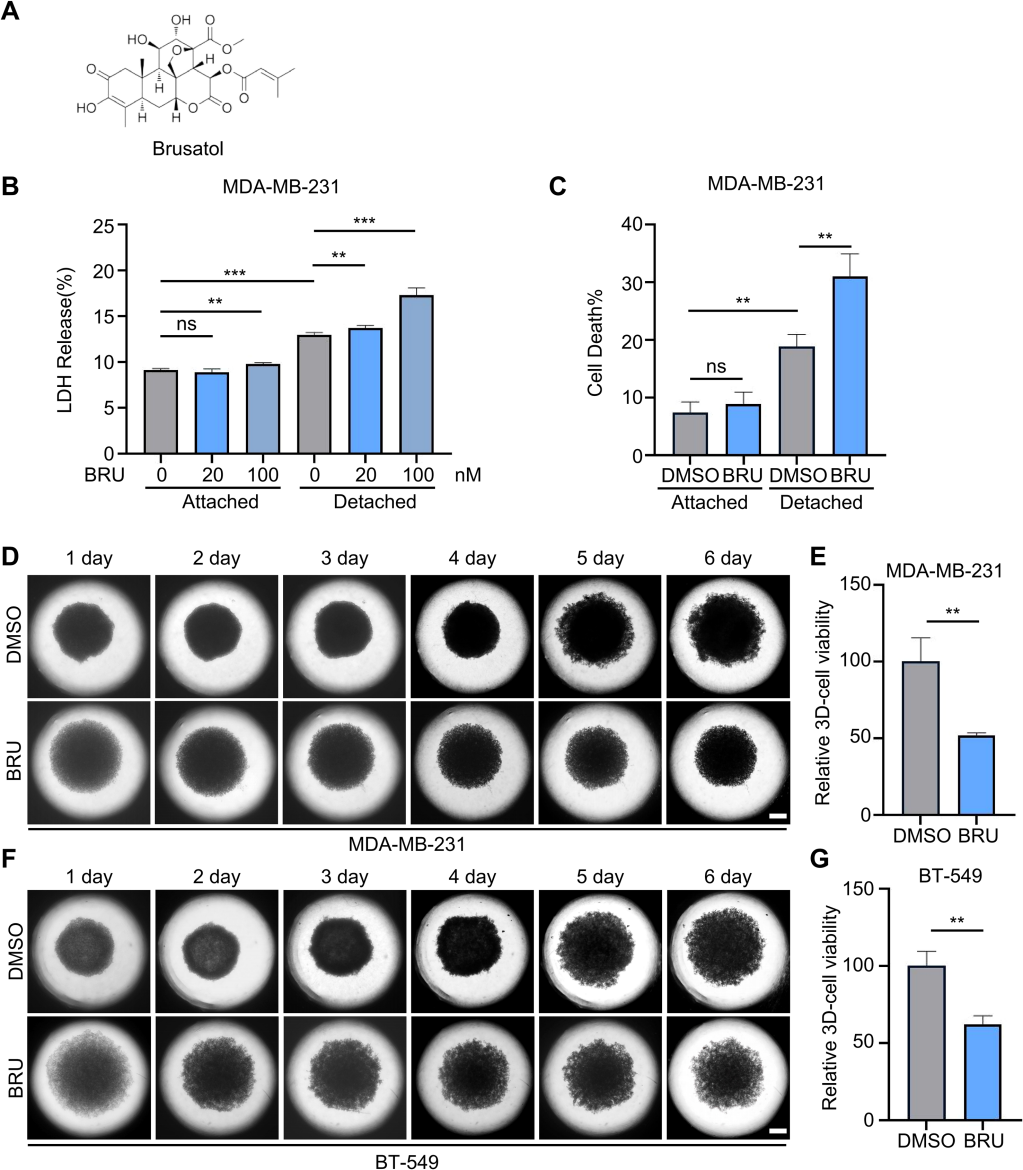
Brusatol promotes TNBC cell death under ECM-detached conditions. **(A)** Scheme of BRU effecting tumor cells under detachment conditions. **(B)** LDH release level of DMSO or BRU-treated (20 or 100nM) MDA-MB-231 under matrix-attached or matrix-detached conditions for 48 hours. **(C)** Mortality rate of MDA-MB-231 cells to BRU (100nM, 36 hours) were analyzed by trypan blue staining. **(D)** Representative images of MDA-MB-231 3D tumor spheroids incubated with DMSO or BRU(100nM) on different days. Scale bars, 20μm. **(D, F)** Representative images of MDA-MB-231 **(D)** and BT-549 **(F)** 3D tumor spheroids incubated with DMSO or BRU(100nM) on different days. Scale, 20 μm. **(E, G)** Relative cell viability of MDA-MB-231 **(E)** and BT-549 **(G)** 3D tumor spheroids on day 6. For **(B, C, E, G)** data were presented as mean ± SD; n=3 biologically independent samples; Two-tailed Student’s t-tests; ***, p< 0.001; ***, p< 0.001; **, p< 0.01; *, p< 0.05; ns, no significance.

Brusatol (BRU) (**Figure 1A**) has been shown to inhibit the proliferation of cancer cells under ECM-attached conditions, suggesting promising anti-tumor activity (32). However, its effect on ECM-detached cells and metastasis remains unexplored. To investigate this, poly-HEMA-coated plates were used to simulate ECM detachment. The impact of BRU on cell membrane integrity in MDA-MB-231 cells was assessed using an LDH release assay, while cell death was evaluated via trypan blue staining. Both assays demonstrated increased LDH release and cell death in MDA-MB-231 detached cells, compared to ECM-attached cells (**Figures 1B, C**). Notably, BRU treatment further enhanced extracellular LDH activity in matrix-detached cells in a concentration-dependent manner (**Figure 1B**). Trypan blue staining revealed that BRU significantly increased the death rate of ECM-detached cells, yet there was no obvious different from DMSO treatment group under ECM-attached conditions (**Figure 1C**). Notably, DMSO treatment alone also resulted in a higher cell death in ECM-detached conditions compared to attached conditions, suggesting the inherent fragility of cells upon matrix detachment (**Figure 1C**).

To further investigate the effects of BRU, we examined the morphological characteristics of tumor spheroids and evaluated cell viability in 3D tumor cultures. During the first 3 days, BRU-treated spheroids were larger than their corresponding controls, indicating that no proper connection between cells was formed (**Figures 1D, F**). Subsequently, the growth of tumor spheroids was restrained by BRU treatment (**Figures 1D, F**). By day 6, the cell viability of BRU-treated spheroids was reduced by 40-50% compared to the control group (**Figures 1E, G**). Collectively, these results suggest that BRU promotes cell death and inhibits the proliferation of TNBC cells under ECM-detached conditions.

### 3.2 Brusatol inhibits TNBC metastasis

**Figure 2:**
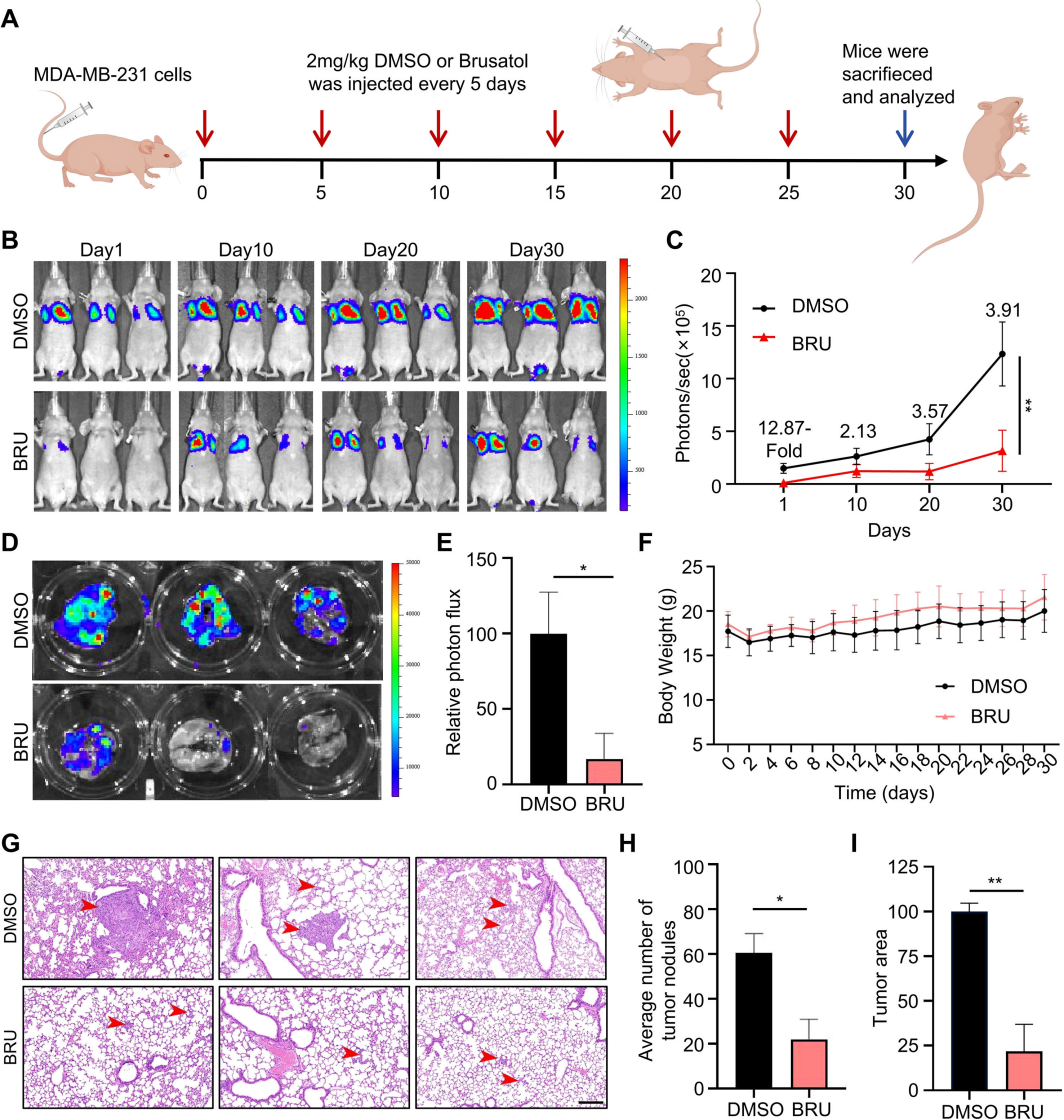
Brusatol inhibits metastatic colonization of TNBC. **(A)** Schematic illustration of the metastasis-inhibition study and general treatment procedure. The blue arrow refers to mice euthanized and lungs removed on day 30. The red arrows indicate the position representing the dosing time and frequency. **(B)** Bioluminescent images of representative mice (n=3) at days 1, 10, 20, and 30 post tail vein injection. **(C)** Quantification of metastasis in lungs of mice from **(B, D)***Ex vivo* imaging of lungs. At the end, mice were sacrificed and the lungs were excised for *ex vivo* bioluminescence imaging. **(E)** Quantification of metastasis in lungs of mice from **(D, F)** Body weight change curves of mice from day 0 to day 30. **(G)** Representative tumor images by HE staining. Red arrows indicate tumors. Three pictures were taken and analyzed per mouse. Scale bars, 200μm. **(H)** Quantification of metastasis numbers in the maximal section of lung tissue. **(I)** Quantification of tumor areas. All data were presented as mean ± SD. For **(C, F)** data, statistical significance was assessed using Two–way ANOVA. For **(E, H, I)** data, statistical significance was assessed using two-tailed Student’s t-tests; **p< 0.01; *p< 0.05.

Given that BRU promotes cell death in suspension TNBC cells, we further evaluated its anti-metastatic effects *in vivo*. A mouse model of lung metastasis was generated by intravenously injecting luciferase-expressing MDA-MB-231 cells into nude mice. Following tail vein injection, mice were randomized into two groups and treated intraperitoneally with either DMSO or BRU (2 mg/kg) six times throughout the study (**Figure 2A**). Bioluminescence imaging of xenografts was performed every 10 days using an IVIS system. On day 1, bioluminescence in the control group was 12-fold higher than that in the BRU-treated group. By day 10, this difference had decreased, with the control group exhibiting 2-fold higher signal, suggesting that BRU delays the colonization of ECM-detached cells in the lungs. By day 20 and 30, BRU treatment significantly inhibited the growth of lung-colonized tumors (**Figures 2B, C**).

Next, we removed the lungs of mice and performed *ex vivo* bioluminescence experiments. The quantitative analysis showed an 80% reduction in the luminescence in the BRU-treated group compared to controls (**Figures 2D, E**). Importantly, no significant loss in body weight was observed in BRU-treated mice during the treatment period (**Figure 2F**), indicating that BRU was well-tolerated within the scope of this study. Furthermore, histological analysis of lung tissues revealed that the control group developed numerous large metastatic lesions, while the BRU-treated group exhibited significantly fewer and smaller metastases (**Figures 2G–I**). The sustained suppression of bioluminescent signal at later time points (**Figure 2C**) and the reduction in both the number and size of lung nodules (**Figures 2H, I**) indicate that BRU not only reduces the initial load of circulating tumor cells but also impedes the colonization of ECM-detached cells in the lungs. In conclusion, these findings demonstrate that BRU effectively inhibits TNBC metastasis *in vivo*.

### 3.3 Brusatol induces metabolic reprogramming, particularly through the PPP

**Figure 3:**
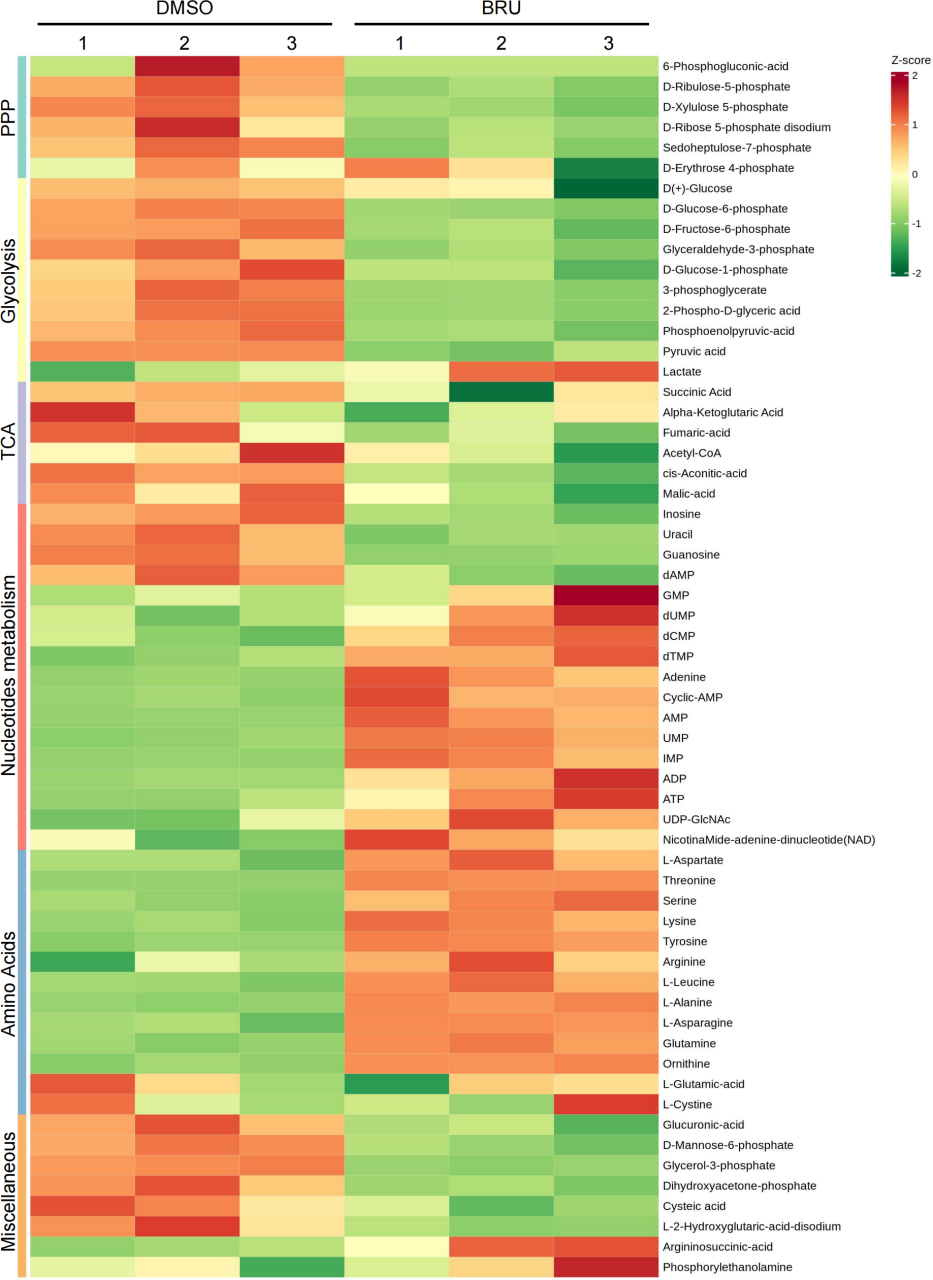
Under ECM-detached conditions, Brusatol broadly changes metabolic reprogramming and particularly through PPP. **(A)** Heatmap showing levels of targeted metabolomics in DMSO or BRU-treated (20 nM) MDA-MB-231 cells upon matrix detachment for 12 hours. The levels of metabolites were normalized by the total amount of protein in each sample. Heatmap was performed using the Metware Cloud, a freeonline platform for data analysis. CoA, coenzyme A; dAMP, deoxyadenosine monophosphate; GDP, guanosine-diphosphate; dUMP, deoxyuridine monophosphate; dCMP, deoxycytidine monophosphate; dTMP, deoxythymidine monophosphate; AMP, adenosine 5′-monophosphate; UMP, uridine 5′-monophosphate; IMP, inosine 5′-monophosphate; ADP, adenosine 5′-diphosphate; ATP, adenosine triphosphate; UDP-GlcNAc, uridine-diphosphate- N-acetylglucosamine.

To investigate the metabolic pathways affected by BRU, LC-high-resolution MS (LC-HRMS)-based metabolomics was performed on MDA-MB-231 cells cultured in suspension. ECM detachment is known to cause a substantial reduction in glucose uptake, and this reduction was further exacerbated by BRU treatment (**Figure 3A**). Notably, metabolomics analysis revealed a significant decrease in intermediates of the PPP (most noticeable change), glycolysis, and tricarboxylic acid (TCA) cycle, including D-Glucose-6-phosphate, 6-Phosphogluconic-acid, D-Xylulose-5-phosphate and 3-phosphoglycerate, upon BRU treatment.

Interestingly, BRU treatment led to an accumulation of D-Erythrose-4-phosphate and lactate (**Figure 3A**). Meanwhile, the intracellular levels of acetyl-CoA were reduced following BRU treatment, suggesting that fatty acid oxidation was also inhibited. Despite the reduction of Ribose-5-phosphate (a nucleotide precursor), the metabolic flux toward nucleotide synthesis was elevated, likely due to compensatory reprogramming. In addition, amino acid levels, particularly glutamine, was noticeably increased. Glutamine metabolism provides nitrogen for amino acid and nucleotide biosynthesis and serves as a carbon source to replenish the TCA cycle, enhancing TNBC cell tolerance (33). Taken together, these findings suggest that BRU broadly affects metabolic reprogramming upon ECM detachment, particularly through inhibiting the PPP.

**Figure 4:**
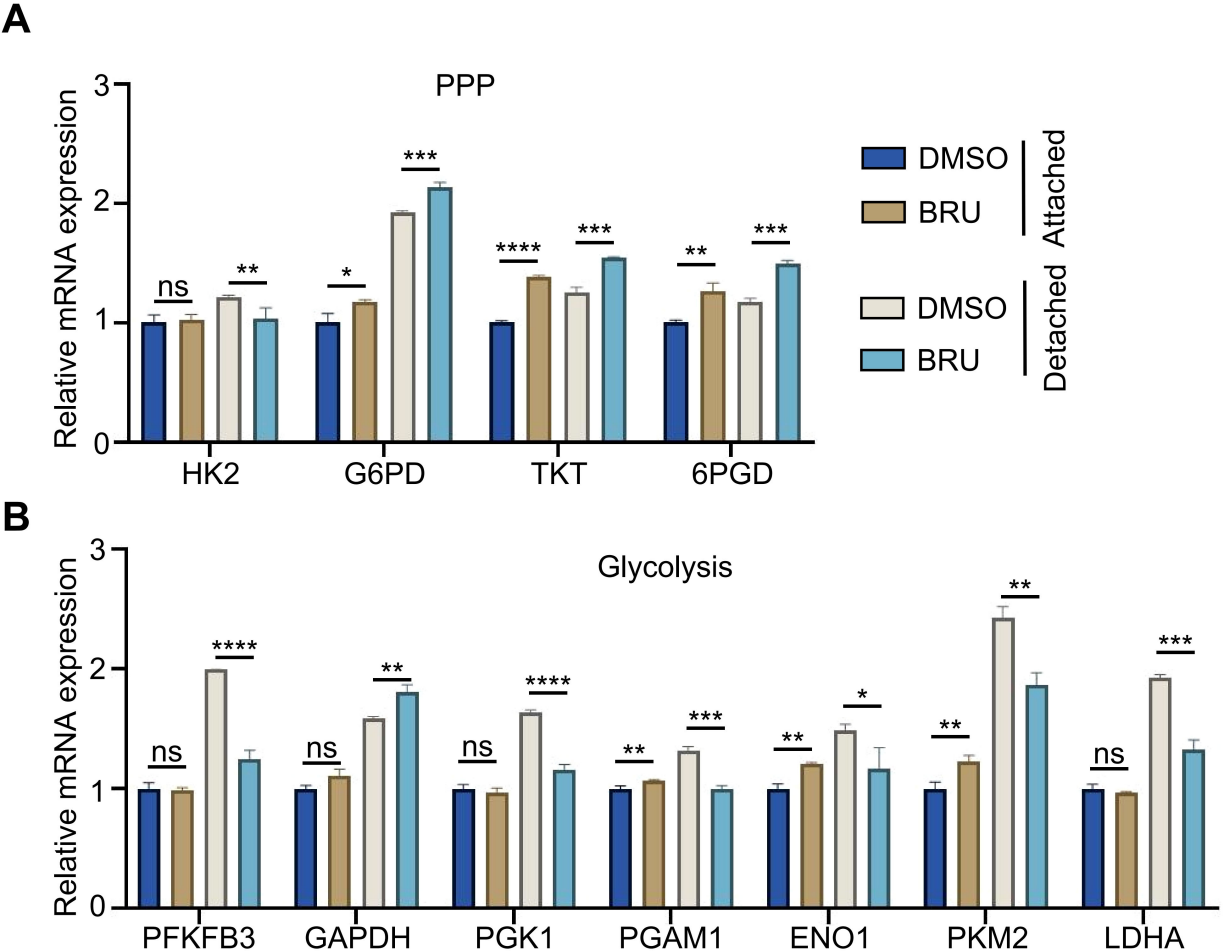
Brusatol effects enzymes in PPP and glycolysis. **(A, B)** The level of mRNA of enzymes associated with PPP **(A)** or glycolysis **(B)** in MDA-MB-231 cells treated with DMSO or 20 nM BRU were detected by RT-qPCR under matrix-attached or matrix-detached conditions for 12 hours. For A and B data, statistical significance was assessed using two-tailed Student’s t-tests; ****p< 0.0001; ***p< 0.001; **p< 0.01; *p< 0.05; ns, no significance.

Given the reduction in metabolite levels, which may be linked to enzymes in the PPP and glycolysis, we further assessed the mRNA expression of key enzymes in these pathways using reverse transcription quantitative PCR (RT qPCR). As expected, ECM-detached MDA-MB-231 cells exhibited higher mRNA levels of most enzymes compared to ECM-attached cells (**Figures 4A, B**). BRU treatment induced only minor changes in mRNA levels in ECM-attached cells (**Figures 4A, B**). Notably, in ECM-attached cells, BRU downregulated several glycolysis-related enzymes, whereas in ECM-detached cells this inhibitory effect was more comprehensive and pronounced (**Figure 4B**). Interestingly, mRNA levels of PPP-related enzymes were generally elevated upon BRU stimulation, with the exception of HK2 (**Figure 4A**). Together, BRU extensively inhibits glycolytic capacity in ECM-detached cells under metabolic stress, compromising their ability to meet basic energy and survival demands, thereby promoting BRU−induced death of ECM-detached cells.

### 3.4 Brusatol impairs redox homeostasis through NADPH depletion

**Figure 5:**
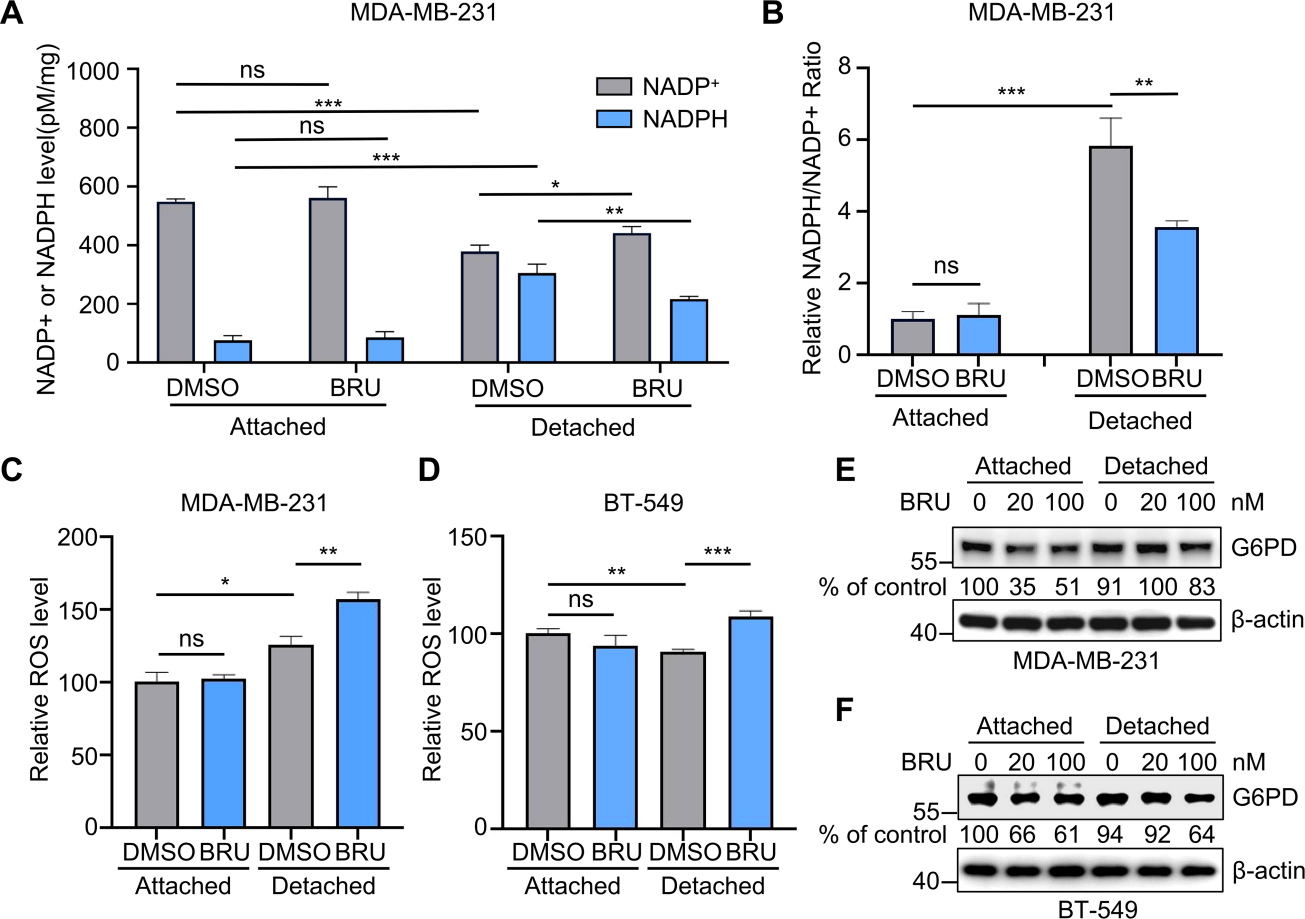
Brusatol reduces NADPH levels and NADPH/NADP^+^ ratio in ECM-detached cells. **(A)** The levels of NADP^+^ and NADPH (normalized by total protein) in DMSO or BRU-treated (20nM) MDA-MB-231 cells under matrix-attached or matrix-detached conditions for 12 hours. **(B)** Relative NADPH/NADP^+^ ratio was calculated from **(A, C, D)** The level of ROS in MDA-MB-231 cells **(C)** or BT-549 cells **(D)** treated with DMSO or 20 nM BRU were detected by Flow cytometer under matrix-attached or matrix-detached conditions for 12 hours. **(E, F)** The expression of G6PD in MDA-MB-231 cells **(E)** and BT-549 **(F)** treated with DMSO or BRU (20 or 100nM) under matrix-attached or matrix-detached conditions for12 hours. For **(A-D)** data were presented as mean ± SD; n=3 biologically independent samples; Two-tailed Student’s t-tests; ***p< 0.001; **p< 0.01; *p< 0.05, ns, no significance.

To elucidate the mechanism by which BRU impacts the PPP in ECM-detached cells, we next assessed its effects on NADPH levels and the NADPH/NADP^+^ ratio. As expected, ECM-detached MDA-MB-231 cells in DMSO-treated exhibited significantly elevated NADPH levels and NADPH/NADP^+^ ratio compared to attached cells (**Figures 5A, B**). BRU specifically reversed this adaptation in ECM-detached cells, significantly reducing both NADPH levels and the NADPH/NADP^+^ ratio, while showing minimal effect in attached cells (**Figures 5A, B**).This BRU-induced depletion of the primary cellular reductant prompted us to assess functional redox status.We performed flow cytometry using DCFH-DA to measure ROS level, which showed that BRU treatment triggered a marked increase in intracellular ROS levels specifically in ECM-detached cells of both MDA-MB-231 cells and BT-549 cells, with no significant change in ECM-attached cells (**Figures 5C, D**).These data directly link PPP inhibition and NADPH depletion to redox stress in the target cells. These findings indicate that BRU exacerbates redox stress in ECM-detached TNBC cells, thereby promoting cell death and inhibiting metastasis.

As a rate-limiting enzyme in the PPP, glucose-6-phosphate dehydrogenase (G6PD) regenerates NADPH from NADP^+^ and regulates oxidative stress through both short- and long-term mechanisms. Previous studies demonstrated that Nrf2 promotes G6PD expression by downregulating TAP73 under ECM-detachment conditions (22). In our study, however, the Nrf2 inhibitor BRU did not reduce G6PD mRNA levels (**Figure 4B**). We therefore assessed G6PD protein levels under both ECM-attached and ECM-detached conditions. Western blot analysis and densitometric quantification indicated a decrease in G6PD protein levels following BRU treatment in both MDA-MB-231 and BT-549 cells, under both attached and detached conditions (**Figures 5E, F**). Notably, a decreasing trend in G6PD protein was observed, particularly at 100 nM in ECM-detached cells. This suggests that the inhibition of PPP activity and NADPH production by BRU may be partly attributable to a reduction in G6PD expression. Taken together, these findings demonstrate that BRU disrupts redox homeostasis in ECM-detached TNBC cells by inhibiting the PPP and depleting NADPH, leading to lethal oxidative stress. While a reduction in G6PD protein was noted at higher BRU concentrations in detached cells, the concurrent upregulation of G6PD mRNA suggests that transcriptional repression is not the only driver of PPP inhibition. Therefore, the mechanism likely involves a combination of partial constraint on G6PD protein abundance and direct or post-translational inhibition of its enzymatic activity, rather than a purely expression-based suppression of the PPP.

## 4 Discussion

Over the past few decades, Chinese herbal medicine and natural compounds have gained substantial attention for cancer therapy due to their low cost, minimal toxicity, ability to reverse drug resistance and promising anti-tumor efficacy. Herbal extracts such as resveratrol (34, 35), curcumol (36, 37), ginsenosides (38, 39) and BRU (29) have demonstrated significant anti-tumor and anti-metastasis effects in various cancer therapy. However, most studies have focused on EMT, tumor cell invasion and migration (37, 40). In this study, we investigated the anti-tumor effects of BRU under ECM-detachment conditions, a persistent state during metastasis. Our findings demonstrated that BRU significantly promotes the death of suspension cells, delays the colonization of circulating tumor cells and inhibits TNBC metastasis. As supported by our *in vitro* findings, the continued and enhanced suppression at later time points, along with the reduction in both the number and size of lung nodules, indicates that BRU also impairs the survival or progressive outgrowth of early-established micrometastases. This suggests that the metabolic vulnerability conferred by PPP dependence targeted by BRU may persist in tumor cells during the initial phases of colonization. Of note, we explored the impact of BRU on tumor cell metabolism, providing the application of BRU in tumor metastasis by targeting metabolism.

Reactive oxygen species (ROS) are byproducts of aerobic metabolism that play essential roles in physiological processes such as signal transduction and protein regulation (41, 42). However, excessive ROS levels can damage DNA, lipid, proteins and even organelle, ultimately leading to cell death (43–45). Accumulating evidence indicates that tumor cells experience overproduction of ROS due to intrinsic and extrinsic factors, such as hypoxia, peroxisomal dysfunction and anchorage-independent growth (7). During rapid proliferation at the primary tumor site and especially during metastasis, ECM-detached tumor cells depend on various defense systems to maintain ROS levels below the lethal threshold (46). These systems include the glutathione and peroxiredoxin pathways, of which NADPH is a universal reducing equivalent (47). Based on these insights, we speculated that BRU promotes cell death in EMC-detached TNBC cell by inhibiting NADPH generation, impairing antioxidant defenses and elevating the level of ROS.

Nrf2, a master regulator of the oxidative stress response, activates the expression of numerous antioxidant and detoxification genes, including G6PD, IDH1/IDH2 (48, 49). It has been shown that Nrf2 inhibition could suppress glycolysis by downregulating glycolysis-related genes, such as GLUT1, HK2, LDHA, and PDK1 (50, 51). In acute myeloid leukemia (AML), BRU has been reported to elicit an anti-AML effect by inhibiting glycolysis and G6PD (52). Similarly, Zhang et al. demonstrated Nrf2 inhibition in breast cancer cells reduced G6PD expression and suppressed PPP and inhibited tumor proliferation and migration (53). In contrast, our study found that BRU treatment under ECM-detached conditions did not lower the mRNA level of G6PD but decreased its protein level, suggesting that G6PD regulation may involve alternative pathways. Therefore, we hypothesize that the inhibition of PPP by BRU in ECM-detached TNBC cells is primarily dependent on the reduction of glucose uptake.

Although targeting altered tumor metabolism is an emerging therapeutic strategy for cancer treatment, its progress therapeutically in the past decade has been limited (54). For example, 2-deoxyglucose (2-DG), a glucose mimetic and competitive inhibitor of hexokinase 2 (HK2) in glycolysis, prevents the production of glucose-6-phosphate (G6P) from glucose, leading to ATP depletion and cell death (55). While 2-DG showed promising results in preclinical studies and advanced to phase I/II clinical trials for the treatment of solid tumors and hormone-refractory prostate cancer, its development was ultimately halted due to limited efficacy in tumor growth inhibition and significant toxicities (56). These challenges highlight the need for novel agents that target tumor metabolism more effectively.

In our study, BRU was found to significantly suppress the flux of PPP, glycolysis, and TCA cycle in ECM-detached TNBC cells while inducing a compensatory increase in glutamine metabolism and ATP levels. This observation is consistent with previous findings that targeting glucose metabolism alone can drive tumor cells to become increasingly dependent on glutaminolysis for survival (57). This metabolic plasticity is driven by the inherent genetic and epigenetic instability of cancer cells, which allows them to rapidly adapt to metabolic stress (58, 59). Despite the inhibition of PPP, glycolysis and TCA cycle by BRU, the extent of cell death in ECM-detached TNBC cells remained insufficient for cancer therapy. Recent studies have shown that the simultaneous blockade of glutamine metabolism with CB-839 and inhibition of glycolysis with 3-BP effectively reduce tumor lesions, while neither drug alone did (60). Based on these findings, we propose that combining BRU with glutaminolysis inhibitors may enhance its therapeutic efficacy in targeting TNBC metabolism. Such combination strategies may help overcome the metabolic adaptability of tumor cells and improve treatment outcomes.

It should be noted that this study has certain limitations. The *in vivo* metastasis experiments employed a modest sample size (n=3 per group). While pronounced anti-metastatic effect of BRU in our pilot investigation were observed, future studies with larger animal cohorts are warranted to further explore the therapeutic window and potential combination strategies of BRU in metastatic TNBC.A limitation of this study is that lung metastases were quantified based on histological morphology in H&E-stained sections. Although the experimental model strongly supports these as human tumor-derived lesions, future studies employing immunohistochemistry with human-specific markers could provide even more precise quantification.

In conclusion, the present study demonstrated that BRU inhibits TNBC metastasis by altering metabolic reprogramming, including the inhibition of glycolysis, the PPP, and the TCA cycle. These findings highlight the potential of BRU as a promising agent for targeted metabolic therapy and suggest that its combination with glutaminolysis inhibitors could further enhance therapeutic efficacy.

## 5 Conclusions

**Figure 6:**
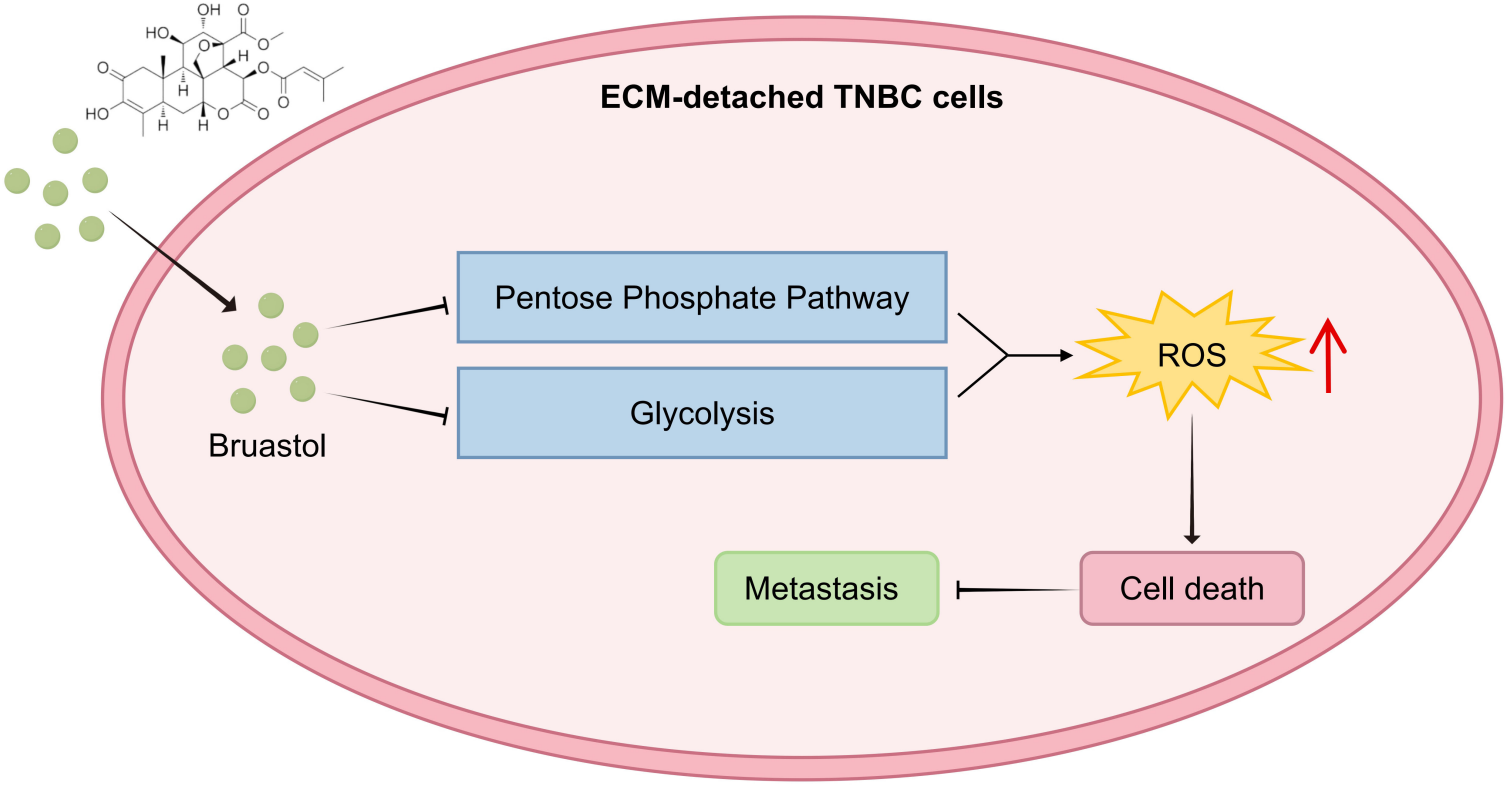
Scheme of BRU promoting cell death in ECM-detached TNBC cells through metabolic regulation. BRU can increase ROS by inhibiting glycolysis and PPP, and promote the death of ECM-detached TNBC cells, which reduced the colonization of secondary tumors.

In our study, we demonstrate that the natural compound Brusatol (BRU) causes detached cells that rely on the pentose phosphate pathway (PPP) to fall into substrate starvation by limiting glucose-6-phosphate (G6P). Although the cells attempt to compensate by upregulating the expression of key PPP enzymes such as G6PD and 6PGD, this response ultimately fails, resulting in NADPH depletion, accumulation of reactive oxygen species (ROS), and irreversible oxidative stress (**Figure 6**). These findings suggest that BRU induces cell death in ECM-detached TNBC cells by metabolic reprogramming, depleting NADPH, and consequently disrupting redox homeostasis and inhibiting the metastatic potential of ECM-detached TNBC cells. Thus, our study represents a promising therapeutic agent for metastatic breast cancer, also providing novel mechanistic insight into the therapeutic against metastatic breast cancer.

## Data Availability

The raw data supporting the conclusions of this article will be made available by the authors, without undue reservation.
